# Big Trajectory Data Mining: A Survey of Methods, Applications, and Services

**DOI:** 10.3390/s20164571

**Published:** 2020-08-14

**Authors:** Di Wang, Tomio Miwa, Takayuki Morikawa

**Affiliations:** 1 Department of Civil and Environmental Engineering, Nagoya University, Nagoya 464-8603, Japan; 2 Institute of Materials and Systems for Sustainability, Nagoya University, Nagoya 464-8603, Japan; miwa@nagoya-u.jp; 3 Institute of Innovation for Future Society, Nagoya University, Nagoya 464-8603, Japan; morikawa@nagoya-u.jp

**Keywords:** trajectory data, data mining, urban dynamics, human mobility, travel pattern

## Abstract

The increasingly wide usage of smart infrastructure and location-aware terminals has helped increase the availability of trajectory data with rich spatiotemporal information. The development of data mining and analysis methods has allowed researchers to use these trajectory datasets to identify urban reality (e.g., citizens’ collective behavior) in order to solve urban problems in transportation, environment, public security, etc. However, existing studies in this field have been relatively isolated, and an integrated and comprehensive review is lacking the problems that have been tackled, methods that have been tested, and services that have been generated from existing research. In this paper, we first discuss the relationships among the prevailing trajectory mining methods and then, classify the applications of trajectory data into three major groups: social dynamics, traffic dynamics, and operational dynamics. Finally, we briefly discuss the services that can be developed from studies in this field. Practical implications are also delivered for participants in trajectory data mining. With a focus on relevance and association, our review is aimed at inspiring researchers to identify gaps among tested methods and guiding data analysts and planners to select the most suitable methods for specific problems.

## 1. Introduction

The development of information and communications technology (ICT) and the proliferation of smart cities have generated tremendous volumes of data comprising specific geographic locations and corresponding time stamps [1]. The Internet of Things (IoT) comprising web-enabled smart devices using built-in sensors [2], radiofrequency identification (RFID), automated fare collection (AFC) systems, the Global Positioning System (GPS), Global System for Mobile Communications (GSM) beacons, and social networks provide abundant trajectory information for researchers to observe urban dynamics on a round-the-clock basis [3]. These trajectory datasets have demonstrated significant academic and practical value; they have been mined and analyzed by researchers to develop solutions for a wide range of emerging but important research questions in fields such as transportation, urban planning, abnormity and violation detection, and environmental protection [3,4,5,6].

Diverse methods have been utilized for analysis; they can be classified as statistical, visual, computational, or a combination of these [3], and they have been tested in many related planning, transportation or geographical studies. However, mining methods are continuously being developed as new and diverse application issues arise [3]. Hence, an integrated survey is urgently needed on the application issues regarding trajectory data and the corresponding mining methods applicable to these issues. Such a survey will help other researchers identify problems, discover methodological gaps, and further develop new methodologies more rationally and efficiently.

The core objective of this paper is to review the methods and applications of trajectory data mining, as well as services that harness these methods to specific urban issues. The rest of the paper is structured as follows. In Section 2, a survey of similar literature reviews focusing on trajectory data mining is presented, which is used for developing research questions. Section 3 briefly elaborates the methodology applied for conducting this research. Section 4 offers an overview on the concept of trajectory data and its classifications. Section 5 discusses mining methods for trajectory data, while Section 6 presents potential applications of these methods as well as the problem-solution mapping relationship. Section 7 reviews the services that are supported by this mapping relationship. Section 8 presents a series of open discussions regarding the practical implications of trajectory data mining. Section 9 concludes this review and presents an outlook for future research.

## 2. Research Questions

Data mining, also popularly referred to as “knowledge discovery” [7,8], is an important process that extracts useful information from huge datasets. Since the emergence of data mining, its methods and applications have been widely investigated in the general data mining domain, as indicated in numerous literature reviews from early stages. For example, surveys of data mining methods for classic relational and transactional data can be found in Fayyad et al. [7] and Han et al. [9], which investigate the general concepts of data mining, and the fundamental techniques for preprocessing, clustering, classification, outlier identification, etc. Beyond this, some scholars (e.g., Mennis [10] and Miller [11]) reviewed theoretical and applied research in spatial and geographic data mining. Such research essentially derives from those in the general data mining domain, with methods specifically adapted to address spatial peculiarities, such as spatial correlation rules and spatial–non-spatial association. These mining tasks present certain rudiments for movement data research, but the reviews fail to consider the temporal dimension that is immanent in trajectory data.

Review papers that focus solely on trajectory data mining rarely follow a complete application-driven framework. Kong et al. [12] categorized trajectory data into explicit trajectory data and implicit trajectory data according to the degree of data structured, and introduced the “applications” of trajectory data from travel behavior, travel patterns, and other aspects. Their review contributed to the classification of multi-source heterogeneous trajectory data, but confused trajectory data mining methods with application issues, as well as practical services. Zheng [3] developed a profound survey on the techniques concerned with different stages of trajectory data mining, following a road map from the derivation of trajectories, to the preprocessing and management of trajectory data, and to the mining tasks such as trajectory pattern mining, trajectory classification, and abnormality detection. This review technically explored the approaches to adapt the existing methods in the general data mining domain to deal with emerging trajectory data, yet lacked the association between practical problems and methodological bases. For this reason, it contributes more to the community of data science than to a broad range of disciplines.

Andrienko et al. [13] developed a taxonomy describing the possible types of information that could be extracted from trajectory data and the respective types of analytical tasks in a systematic way. This taxonomy considers three fundamental sets, i.e., space, time, and objects, and distinguishes tasks according to the relations among the elements involved in each set. Andrienko et al. [13] also discriminated generic classes of analytical techniques, including visualization, data transformation, computational analysis methods, etc., and linked the types of tasks to the classes of techniques that could support fulfilling them. This work helps to match generic approaches with specific tasks in the field of trajectory data mining. However, it merely focuses on the methods developed from GIS-based visual analytics. By contrast, the contribution of our work breaks through this limitation, and moves into a broader field of computational analysis.

From a pure application perspective, Castro et al. [6] surveyed the existing research on mining taxi GPS traces, and grouped the surveyed work into three categories: social dynamics, which studies the collective behavior or movement patterns of a city’s pollution; traffic dynamics, which studies the resulting flow of the population through the city’s road network; operational dynamics, which learns from taxi drivers’ knowledge of the city. This categorization method is more intuitionistic than Andrienko et al. [13] and has been widely referenced by researchers in the field of taxi trajectory mining, for example in [14,15,16]. However, Castro et al. [6] only considered the application of taxi trajectories. Besides, the matching relationship between fundamental mining methods and practical applications were not clearly elaborated. Our paper extends their work by considering beyond a specific kind of trajectory, meanwhile focusing more on the application issues in each category as well as their corresponding solutions.

Based on the review of similar research, the following gaps are identified. First, the exponential growth of ICT has enriched the connotation of trajectory data in recent decades, while some existing review papers are limited to specific kinds of trajectory data, e.g., taxi GPS traces in Castro et al. [6] or photo streams with spatiotemporal tags in Andrienko et al. [13]. In order to acquire a well-rounded understanding towards trajectory data mining, the first research question has been outlined as: What kinds of trajectory data can be utilized nowadays? Following this prerequisite, the major questions forming this application-driven research are delivered naturally: Which mining methods are applied or adapted to deal with trajectory data? What are the up-to-date application issues in trajectory data mining? What are the practical services that can be developed from studies in this field? Besides these obvious questions, there is a core thought that runs through our entire research and distinguishes our research from previous reviews: What is the matching relationship between the mining methods, the application issues that can be solved by these methods, and the practical services that can be derived from these applications?

## 3. Methodology

**Figure 1:**
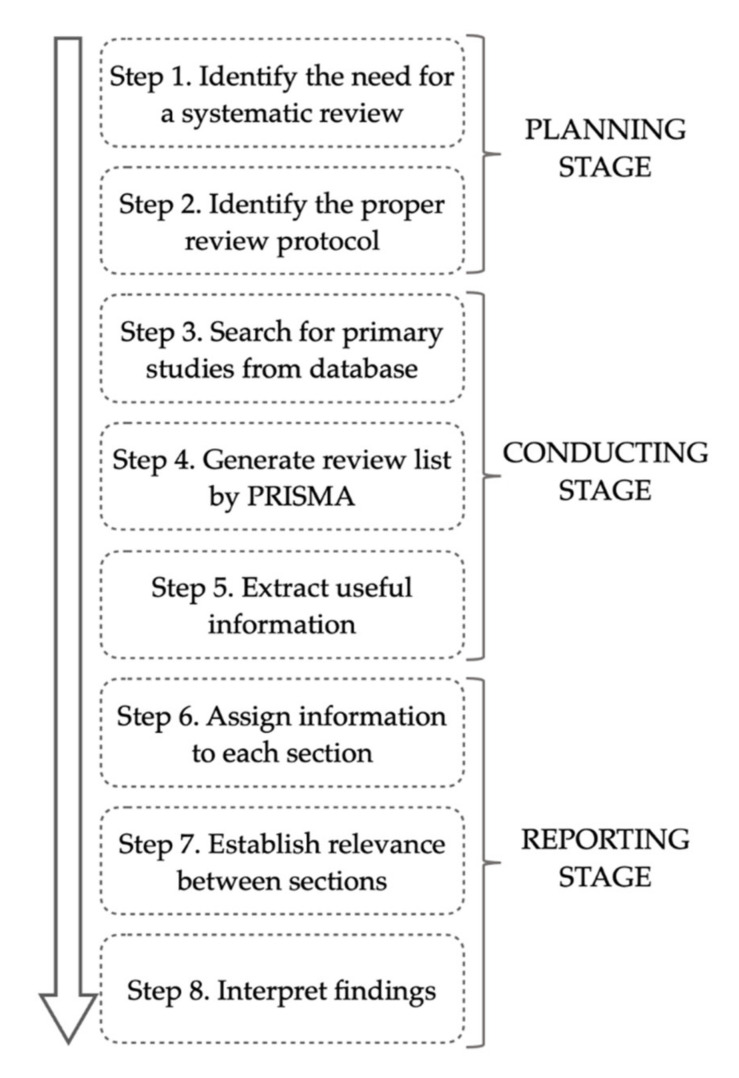
Research steps for the literature review.

In order to answer the research questions proposed above, a systematic literature review (SLR) is performed “with respect to the planning for literature review, the design of search string, sources to be searched, publication inclusion and exclusion criteria, publication quality assessment and the data extraction process” [17]. Following the methodology indicated by Bach et al. [18] and Wahono [19], our research procedure is designed as shown in Figure 1. Since the major sections of our work follow a narrative workflow from trajectory mining methods to applications of these methods and to practical services derived from these applications, the literature for each section may be scattered and independent from each other. Therefore, unlike Bach et al. [18], which focuses on the applications of textual mining specifically in the financial sector, the literature reviewed in our research is evaluated by manual judgement, rather than the bibliometric software they indicated. The relevance between the literature in each section is also established through manual analysis.

Based on the analysis of existing literature reviews that are concerned with trajectory mining as presented above, the need for a systematic review and its research questions are identified (Step 1). Following the established review protocol in Moher et al. [20] (Step 2), the materials for this research are acquired by searching publications using the search string “trajectory data mining”, for the period from 2004, in the Web of Science Core Collection database (Step 3), and then, are selected according to the Preferred Reporting Items for Systematic Reviews and Meta-Analyses (PRISMA) standard [20] for writing SLRs to form our review list (Step 4). Useful information is manually extracted from the selected literature (Step 5) and assigned to each major section that corresponds to research questions (Step 6). The relevance between the information assigned to each major section is established based on manual analysis (Step 7), so as to interpret the matching relationship between trajectory mining methods, application issues, and practical services (Step 8). During the reviewing process, additional literature is tracked and included by snowballing approaches [20], in order to fulfil our research needs. Thus, the reference list of this paper is longer than the original review list generated by PRISMA.

## 4. Trajectory Data

What does the concept of a “trajectory” mean in the field of data mining? According to Zheng et al. [3,21], it is a trace generated by a moving object within a certain spatiotemporal context and is generally represented by a series of chronologically ordered points. In other words, trajectory data are essentially a sequence of spatial points ordered by timestamps and generally carry some descriptive information in addition to basic spatiotemporal messages. Therefore, a piece of trajectory data can be described as *TR* = <*P*_1_, *P*_2_, …, *P*_n_>, where *P_n_* = (*ID_n_*, *X_n_*, *Y_n_*, *T_n_*, *A_n_*) is the *n*th trajectory point; *ID_n_* is the identifier; (*X_n_*, *Y_n_*) is the location of *P_n_* in the specific coordinate system (i.e., natural geographic coordinate system or self-built coordinate system); *T_n_* is the timestamp of the point (i.e., the moment when *P_n_* is event-triggered [22] or regularly recorded); *A_n_* is potentially a list of additional descriptive properties for *P_n_* (e.g., instantaneous speed, running direction).

**Figure 2:**
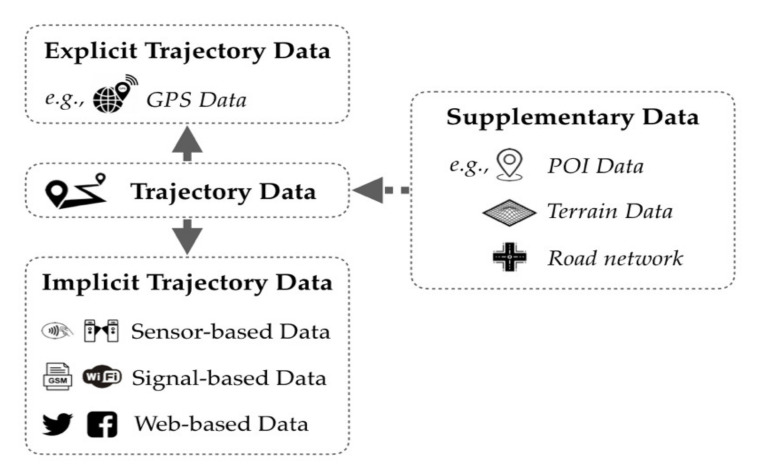
Trajectory data categories [12].

Different categories of trajectory data have been emerging and are being applied with the development of ICT. Renso et al. (2013) [23] distinguished GPS, GSM, and geosocial networks as significant carriers of trajectory data. Soon afterwards, Pelekis and Theodoris (2014) [24] enriched trajectory data sources with RFID and Wi-Fi. These data carriers represent currently existing types of trajectory data, which can be roughly categorized as explicit or implicit [12], as illustrated in Figure 2.

### 4.1. Explicit Trajectory Data

In this paper, explicit trajectory data are defined as a type of well-structured data which directly provide time and location information and have strong spatiotemporal continuity. They are regularly collected by terminal equipment at high (and usually fixed) frequencies, with no need to be triggered by any specific events. For example, trajectory data reported from GPS devices equipped in taxis are an uninterrupted series of spatiotemporal points recorded at fixed time intervals (e.g., 30 s intervals in most Chinese cities [25]). This kind of data is well-structured and contains relatively precise and direct spatiotemporal information along with other data fields, such as data fields indicating altitude, speed, direction, vehicle status, etc. [26].

In such cases, the above trajectory point model can be further evolved into a trajectory segment model: *TR* = <*SubTR*_1_, *SubTR*_2_, …, *SubTR*_n_>, where *SubTR* = (*ID*_sub_, *trP*_1_, *trP*_2_) is the sub-trajectory (i.e., segment) that forms the complete trajectory; *ID*_sub_ is the unique identity of the segment; *trP*_1_ and *trP*_2_ are the adjacent chronologically ordered trajectory points that delineate the segment. The trajectory segment can be obtained by finding the linear difference between *trP*_1_ and *trP*_2_, while the complete trajectory consists of *n* such segments connected end to end in chronological order.

### 4.2. Implicit Trajectory Data

Apart from explicit trajectory data, there exist such data carrying spatial and temporal information. Although they are not the trajectory data we usually think of, trajectory information can be extracted from them after basic data processing operations. This paper refers to such data that do not directly represent trajectory information as implicit trajectory data. In contrast to explicit trajectory data, implicit trajectory data have no definite continuity in time and space. In other words, they are triggered by an event rather than passively recorded. Such an event may refer to a bus or subway check-in, social network sign-in, sensor activation, signal tower reception, etc.; points with spatiotemporal information will not be recorded unless the corresponding event happens.

Unlike explicit trajectory data, which are usually recorded in a structured database format (e.g., Oracle DMP), the storage formats of implicit trajectory data are diverse and unstructured (e.g., text, image, audio, video) because of the variety in data sources and data collectors [21]. Although these forms of data present different properties, they have been applied to deal with similar or correlative issues among common mining methodologies [23,24].

#### 4.2.1. Sensor-Based Trajectory Data

Sensor-based trajectory data (e.g., active fiber composite (AFC) and transit smartcard data [27]) are recorded when an object passes through a sensor. These sensors are mounted at a series of fixed positions and can only be activated at very close distances. Thus, sensor-based data have high spatiotemporal accuracy but weak spatiotemporal continuity, which is limited by the number of sensors.

#### 4.2.2. Signal-Based Trajectory Data

The collection of signal-based trajectory data requires multiple signal projectors (e.g., cell towers, Wi-Fi transmitters, Bluetooth connectors) to be distributed in advance. GSM-based data consist of chronologically ordered sequences of cell identifiers along which the moving object passes. Wi-Fi and Bluetooth-based data comprise temporal sequences of identifiers of access points that have communicated with the moving object [28]. This type of data is more complex than the previous ones and generally contains the device ID, connection/disconnection timestamp, signal strength, etc. Preprocessing is needed to extract trajectory information [29].

#### 4.2.3. Web-Based Trajectory Data

Web-based trajectory data are contained in a geolocalized social network. Recent years have witnessed the rise of social sites/apps (e.g., Twitter, Facebook, Weibo) equipped with geotag functions. In addition to spatiotemporal information, such social network services also carry semantic information regarding specific events, human activities, emergencies, etc. [30]. To some extent, web-based trajectory data are more informative than any other categories because useful knowledge may be extracted from the additional semantic information they are carrying, but they are also more implicit due to the noise they contain and semantic messages that are difficult to process.

### 4.3. Supplementary Data

In most studies, trajectory data do not function alone; they are projected into built environments for better analysis. Such built environment information (e.g., points of interest (POIs), road network, terrain distribution, urban structure), which is usually integrated with trajectory data in data mining applications, is regarded as supplementary data. This kind of data generally exists on or can be extracted [31] from comprehensive digital map platforms.

POI is a major data category that represents the reality of built environments. It literally refers to a specific point that someone finds useful or interesting [32]; POI data contain information for almost all key nodes within an urban area (e.g., locations of buildings concerned with retail, catering, education; administration and locations of facilities related to transportation, communication, and security) [32,33]. The supplementary information from POI data can be used to develop a more reasonable and rational explanation for patterns and behavior detected in trajectories.

Other supplementary data are mainly related to the geographical context of trajectories [34] (e.g., road network, elevation system). In fact, it is quite a natural perception and practice to connect trajectory data with such geographic frameworks because geography is one of the two most remarkable attributes of trajectories (the other is time) [35]. Existing online map platforms (e.g., open-source platforms like OpenStreetMap (OSM) and commercial platforms like Google Map and Baidu Map) can serve as sources for these supplementary datasets [36,37,38].

## 5. Trajectory Data Mining Methods

What does trajectory data mining mean? Similar to the common understanding of general data mining, trajectory data mining means to discover interesting knowledge (e.g., movement patterns, travel behavior, traffic abnormality) from trajectory datasets. Generally, trajectory data mining has two major tasks: description and prediction [3,21]. Description is to interpret human-readable information from massive volumes of trajectories, while prediction is to discover uncharted or prospective values by analyzing existing variables in datasets. These two basic tasks are performed for all applications and services that are related to trajectory data.

With regard to methods for trajectory data mining, this paper focuses more on the methodology or principles rather than listing all specific technical procedures that have been adopted in previous research. We concentrated only on the prevalent methods and tried to ascertain the connections between them. We divided these methods into two categories: first-tier and second-tier methods. The former sorts trajectories directly based on their attributes, while the latter usually contains sequences of first-tier methods, sometimes together with non-mining methods (e.g., statistical or topological), to study the spatiotemporal permutation of trajectories.

### 5.1. First-Tier Trajectory Data Mining Methods

As noted above, first-tier methods classify trajectories directly from cleansed datasets based on their inherent properties. These methods are basic, yet most important in the field of trajectory data mining. The application of first-tier methods is usually followed by a descriptive interpretation of the results, and on many occasions, functions as the preparation for subsequent extended analysis, such as with second-tier methods. Data cleansing is a preprocessing task before first-tier data mining, but we will not discuss it in detail here. Detailed information regarding data cleansing can be found in [39,40,41]. In this article, all of the methods discussed are assumed to be based on cleansed datasets.

#### 5.1.1. Clusterings

Clustering is a first-tier trajectory data mining method. It is an unsupervised learning process that reveals similarities within a trajectory dataset by dividing trajectories into categories (i.e., clusters) according to their properties to indicate homogeneity and heterogeneity [42]. In other words, the movement characteristics of trajectories should be similar within a cluster, while different between clusters. A general clustering approach is to represent each trajectory with a feature vector, and then, measure the similarity between trajectories by calculating the distance between their feature vectors [3]. However, it is not easy to generate feature vectors with a uniform length for different trajectories, since trajectories may vary significantly in terms of length, shape, sampling frequency, point quantity, point order, and many other properties. Besides, it is also difficult to encode the sequential properties of points in a trajectory into its feature vector.

Considering the challenges mentioned above, a series of technical explorations have been done. On the one hand, there are widely accepted clustering algorithms for trajectories which are essentially extensions of classical clustering algorithms [43,44,45] with specific customization on the similarity (or distance) functions to determine cluster membership. A detailed discussion on how these functions are applied can be found in Rokach [45]. Generally, depending on the goal of analysis, similarity (or distance) functions such as similar destination, similar origin, similar direction, or others are utilized to determine which trajectories belong to the same cluster.

On the other hand, there have been efforts to develop trajectory-specific clustering approaches. Many of them are accommodating statistical or probabilistic models for measuring the characteristics of trajectories. For instance, Gaffney and Smyth [46] and Cadez et al. [47] proposed mixed regression model-based approaches to aggregate trajectories likely to be generated by a common representative trajectory with Gaussian noise. The Expectation Maximization (EM) algorithm they proposed clusters trajectories with respect to the overall distance between two entire trajectories. Similarly, Alon et al. [48] abstracted trajectories as sequences of position transitions and utilized a Hidden Markov model (HMM) that best fit the trajectories to select cluster members.

The approaches proposed by Gaffney and Smyth [46], Cadez et al. [47], and Alon et al. [48] are applicable to entire trajectories. In other words, they group similar trajectories as a whole. However, in reality, moving objects rarely move together for an entire path. Besides, discovering common sub-trajectories is also useful in many applications, especially when there are regions of special interest for analysis. To this end, Lee et al. [49] proposed a partition-and-group framework, which partitions an entire trajectory into a set of line segments, and groups similar line segments into a cluster using the Trajectory Hausdorff Distance [50]. A representative trajectory describing the overall movement of the trajectory partitions that belong to a cluster is identified by sweeping a vertical line across the line segments in the direction of the major axis of a cluster.

The clustering approaches mentioned previously are developed for static datasets. They are not suitable for incremental clustering, when trajectory data are received incrementally, e.g., continuous new points reported by a GPS system. Li et al. [51] proposed an incremental clustering framework for trajectories to deal with this situation. The framework has two components: online micro-clustering maintenance and offline macro-clustering creation. For the online part, micro-clusters are incrementally updated when new data are added; for the offline part, when the user requests current clustering results, macro-clustering is performed on the sets of micro-clusters rather than all trajectories over the entire time span. This approach is able to save the computational cost and the storage of received trajectories when processing trajectory data streams.

All the methods discussed in this section are oriented towards trajectories in a free spatial context, i.e., with no road network constraints. Kharrat et al. [52] proposed the NETSCAN algorithm that applies specifically to trajectories that lie on a predefined network. NETSCAN is essentially an extension of classic DBSCAN that first computes dense paths in the network and then, clusters the sub-trajectories similar to the dense paths. Apart from this, there are few studies focused on trajectory clustering in a road network setting, because this task can be easily solved by the combination of map matching and regular trajectory clustering algorithms. Map matching is the process to project trajectories onto a corresponding road network, and meanwhile, attaching road network information to the trajectories. Map matching approaches can be found, for example, in Miwa et al. [53] and Quddus et al. [54].

#### 5.1.2. Classification

Classification differs from clustering because it is a supervised or partially supervised learning process [55]. The classification classes need to be predefined, and a training set of objects needs to be prelabeled with the class that they belong to. For example, a typical case of trajectory classification may be to label each trajectory from a large set with its means of transportation based on a small set of trajectories that have already been labeled. This small set is the training set. Thus, the labeling process (i.e., assigning objects to predefined classes based on the means of transportation) is classification.

A typical trajectory classification algorithm contains two steps. First, it needs to extract a set of discriminative features that can be used to train an existing standard classification model (e.g., logistic regression [56], support vector machine (SVM) [57], decision trees [58], nearest neighbors [59]). This step is to find the trajectory properties that are best suited to defining the various classes of trajectories. Trajectories have many potential useful properties (e.g., transportation means, average speed, time duration, trajectory length), but their discriminative power depends on the type of classes expected. For example, if the taxi fare is a class type, the trajectory length has a stronger discriminative power than the time duration because taxis charge according to mileage rather than time. The second step is to select a proper standard classification model, and then, apply it to the extracted discriminative features.

Several comparative studies have been performed on standard classification models and their corresponding classical classification algorithms [60]. Most of these classical methods can be directly applied to trajectory classification. For example, Bolbol et al. [61] utilized SVMs for transportation mode classification. They first evaluated the discriminative power of several features for six transportation modes (i.e., bus, subway, train, private car, bicycle, and walking) through statistical methods, and identified speed and acceleration as the most discriminative. Second, they applied a standard SVM algorithm to these features to classify trajectory segments. Zheng et al. [62] did similar work, except that that they applied a decision tree-based inference model to the discriminative features for transportation mode classification.

In many situations, trajectories are classified following some preprocessing (e.g., segmentation, clustering, statistical analysis) that prepares the features needed for classification [62,63,64]. For example, Zheng et al. [62] proposed a change point-based segmentation method to partition each complete trajectory into separate segments of different transportation modes; they identified a set of features not affected by differing traffic conditions that could be fed to the inference model. Lee et al. [63] performed trajectory clustering to extract regional and sub-trajectory features for an SVM-based classification model.

### 5.2. Second-Tier Trajectory Data Mining Methods

The first-tier methods presented above are generally used to categorize trajectories. In many cases, they are then followed by second-tier trajectory mining methods, which are used to analyze the spatiotemporal characteristics of the individual trajectories within or between categories that were identified by the first-tier methods. In other words, this is a subsequent processing of the results from the first-tier processing. Many types of methods are available for this stage but the three most versatile are pattern mining, outlier identification, and prediction.

#### 5.2.1. Pattern Mining

Pattern mining concentrates on discovering interesting, significant, or unexpected patterns that exist in databases. It is one of the most fundamental tasks of data mining [65]. Various patterns can be mined (e.g., frequent items, sequential rules, periodic patterns, subgraphs, associations) corresponding to various algorithms for pattern mining (e.g., frequent pattern (FP) growth, a priori, ECLAT) [66,67,68]. These algorithms can be categorized into three types of trajectory pattern mining: periodic, frequent, and collective.

A periodic pattern refers to trajectories periodically executed by a moving object [69,70,71]. For example, it may reflect the regular movement patterns from office staff, which are rather similar each working day. In contrast, a frequent pattern is not focused on such temporally repetitive phenomena of individuals but refers to a specific sequence of places that have been visited by a certain number of moving objects with no specific temporal constraints [66]. A typical example of a frequent pattern is a park itinerary, which is followed by most tourists. A collective pattern is a combination of these two and is performed by groups sharing similar mobility interests both temporally and spatially. In other words, these moving objects travel together [70]. Periodic pattern mining utilizes location sequences as mining criteria. Early-stage approaches [71,72] require the time period to be a specific input in the mining algorithm. They cluster the sequences of locations in each preset time branch and then, iteratively connect the detected frequent sequences to obtain the integral pattern. However, such work to preset the time period involves many uncertainties. For example, the division of time intervals will definitely affect the clustering output, but these effects are difficult to measure. Meanwhile, different time periods may occur as the discovery progresses, but these algorithms are not equipped with dynamic adjustment capabilities. Li et al. [73] developed the Periodica algorithm to overcome these problems by bypassing the time period presetting. Their algorithm selects regions where more trajectory points exist as the reference spots and then, automatically detects the periods in each spot through a combination of Fourier transformation and autocorrelation. These periods are used to discover periodic patterns from location sequences between reference spots. Hierarchy-based clustering with a probability-based distance measurement model is performed on these location sequences. Compared to previous algorithms, Periodica better matches realworld scenarios because the period-setting criteria are unpredictable in principle until the real movement sequences are considered [74].

**Figure 3:**
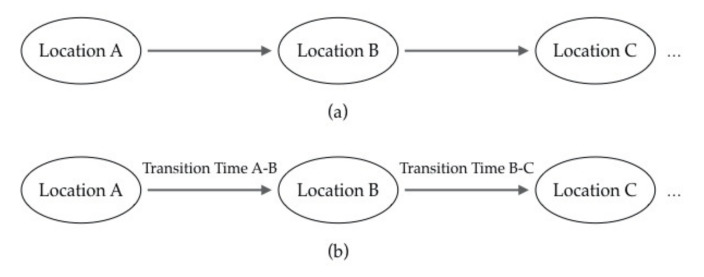
Frequent patterns based on (**a**) spatial sequences and (**b**) spatiotemporal sequences.

Frequent pattern mining focuses on the collective routes or paths that have been frequently traveled by multiple moving objects [66]. Thus, such patterns can be discovered simply by using the spatial features of trajectories [75] (i.e., only the sequences of spatial locations need to be considered). Typical examples include frequent spatiotemporal sequential patterns (FSSP) mining [76] and generalized sequential patterns (GSP) mining [77]. However, some have considered frequent patterns not only as spatial elements but also as temporal elements along spatial trajectories. For example, Giannotti et al. [78,79] defined the T-pattern as an assemblage of individual trajectories sharing the common attribute of visiting the same sequence of locations with similar transition times. There are roughly two scenarios for frequent patterns, as illustrated in Figure 3. For Figure 3a, frequent pattern mining can be based on the clustering methods discussed in Section 5.1.1 or simply by applying statistical analysis [76,77]. For Figure 3b, however, frequent patterns cannot be discovered within one step. Thus, a two-step approach can be used [79,80], which consists of detecting significant regions outside the trajectories and then, performing sequence mining in these regions as a temporally annotated sequence.

**Figure 4:**
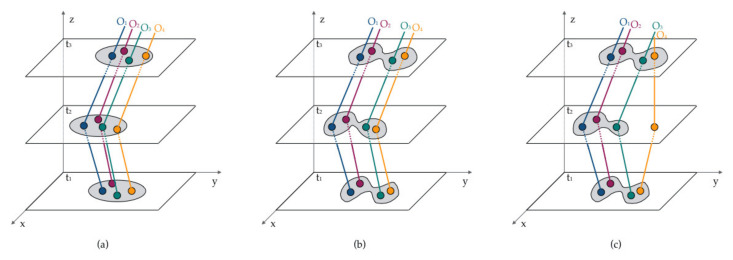
Collective pattern categories: (**a**) flock, (**b**) convoy, and (**c**) swarm [3]. Each image contains three timestamps (i.e., *t*_1_, *t*_2_, *t*_3_) and four moving objects (i.e., *O*_1_, *O*_2_, *O*_3_, *O*_4_).

Collective pattern mining (i.e., group pattern mining) essentially finds movement patterns that have been performed by groups with similar mobility interests [70]; such interests require not only spatial proximity but also time coordination. Considering the spatiotemporal closeness, internal structures, and external performances, group patterns can be roughly categorized into three types, as illustrated in Figure 4: flock, convoy, and swarm [3]. A flock [81,82] refers to a group of at least *o* objects that move together for at least *t* successive timestamps; the positions where these moving objects remain on each time slice can be observed in a disk with a radius *r*. Thus, such patterns can be described with three parameters: *o*, *t*, and *r*. A convoy [83,84] is similar to a flock except for its relaxed requirements for the disk shape. A convoy pattern allows its moving objects to form any disk shape on each time slice as long as the positions can be clustered, usually by density-based clustering with a maximum neighborhood distance *d* and minimum object number *o* [83]. A swarm further relaxes the requirements for a convoy. The timestamps do not need to be successive in this situation; in other words, there does not need to be at least *o* positions that can be clustered on every time slice [85]. A swarm shares similar parameters to the other two, including *o*, *t*, and *d*. However, it also includes the swarm parameter *k*, which indicates the minimum number of time slices on which the collective patterns can be detected. For example, Figure 4c shows a swarm situation where *o* = 4, *t* = 3, and *k* = 2. It is neither a flock nor a convoy because object *O*_4_ breaks away from the group at timestamp *t*_2_. It can be considered a swarm pattern because all four objects can be clustered into one group at timestamps *t*_1_ and *t*_3_. Density-based clustering is the most common method for collective pattern mining, whether it is for flocks [86], convoys [83,87], or swarms [88,89]. In most cases, clustering is the first-tier step, while parameters are checked next to determine which category a collective pattern belongs to.

#### 5.2.2. Outlier Identification

In data mining, outlier identification (i.e., outlier or anomaly detection) involves the detection of rare items, events, or observations that arouse suspicion by differing significantly from the majority of the dataset. Outliers can also be referred to as anomalies, novelties, noise, deviations, and exceptions [90]. For trajectory data, outlier detection involves discriminating trajectories that are barely consistent with the common characteristics of the majority of trajectories [91]. To some extent, it is complementary to the above trajectory mining methods; these methods focus on the homogeneity of the data, while outlier identification is more concerned with the heterogeneity.

Thanks to this complementary relationship, a major methodology for outlier detection is to concentrate on the byproducts of trajectory clustering. Theoretically, trajectories that do not belong to any cluster should be outliers. However, a significant disadvantage of this indirect approach is that it cannot guarantee sufficient differentiation between byproducts. In other words, these byproducts may also show some similarities, although these similarities may not satisfy the clustering criteria of the previous step. Thus, further detection should be performed to distinguish real outliers among these byproducts. This kind of work may fall into a trap of continuous looping.

**Figure 5:**
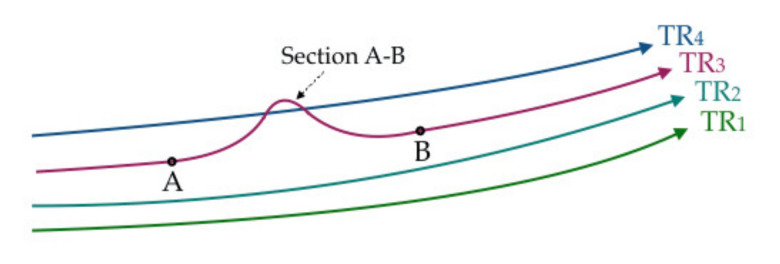
Example of an outlying sub-trajectory: section A–B of trajectory TR_3_ [91].

There have been some attempts at outlier identification from a more direct perspective. One approach is to mine all trajectories for outliers [92,93]. In such cases, each complete trajectory is abstracted to a set of key features (e.g., the spatial coordinates of the start and end points, the values (minimum, maximum, mean) of the directional vectors and velocities). Then, distance-based algorithms, which are usually equipped with a distance function defined as the weighted sum of the differences of the abstracted features [93], are applied to outlier detection. In this situation, the basic unit for mining is the complete trajectory. However, such methodology may not be able to find outlying trajectory sections. For example, Figure 5 clearly shows that section A–B of trajectory TR_3_ is different from its neighboring trajectories (TR_1_, TR_2_, TR_4_), but it may not be distinguished as unusual because the overall behavior of TR_3_ is similar to that of its neighbors. From a mathematical perspective, the significant differences in sub-trajectories may be averaged out over the complete trajectory.

**Figure 6:**
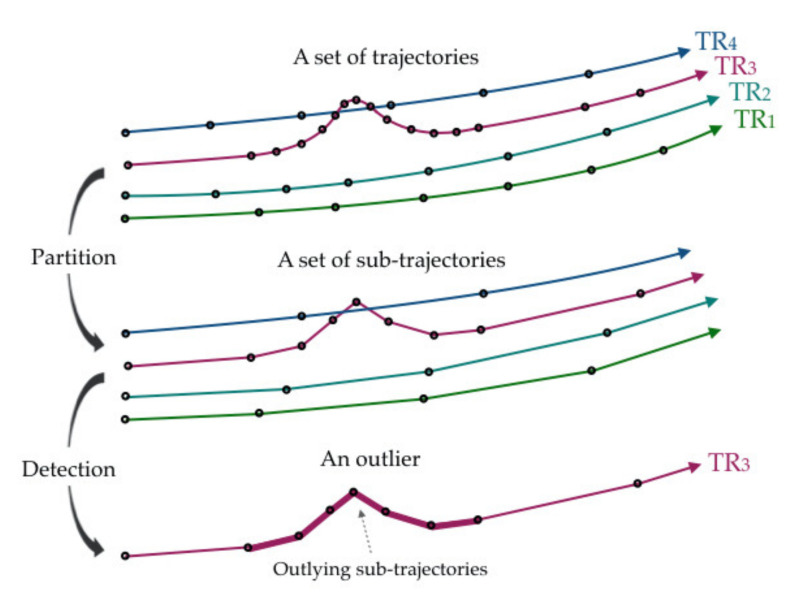
Partition-and-detect framework [91].

Another approach is to focus on the decomposed trajectories [91,94,95]. These methods generally partition each complete trajectory into a set of sub-trajectories and then, detect the outlying sub-trajectories by applying a distance function or clustering approach. Eventually, the complete trajectories that contain outlying sub-trajectories are discriminated as outliers. Lee et al. [91] defined this as a partition-and-detect framework, as demonstrated in Figure 6, and proposed the TRAOD algorithm, which utilizes a hybrid of distance-based and density-based approaches for the second step of outlier detection.

These methods are generally based on clustering and its extensions; in other words, they are unsupervised or semi-supervised learning processes. However, supervised learning approaches are also available for outlier identification; these are typically based on classification. For example, Yuan et al. [95] extracted a set of pre-identified features (i.e., direction, speed, angle, and location) from trajectories to which they then applied distance measures to discriminate anomalies. Li et al. [96] utilized trajectory features to train a two-label classifier model: one label classified normal trajectories while the other classified abnormal ones.

#### 5.2.3. Prediction

In data mining, prediction involves assuming that certain turns of events will occur based on the description of other related data. The prediction itself is calculated from the available data and modeled in accordance with the existing dynamics [97]. There are two approaches to predictions using trajectory data: predicting the future location of a moving object, and predicting its entire route within a road network context. There are three categories of location prediction: (1) based on the dynamics of the moving objects of concern [98,99,100,101], (2) based on the dynamics of other objects (e.g., a set of located users with a social-spatial performance that exceeds IP-based geolocation) [102], and (3) based on both the objects of concern and other objects [103,104,105].

Two major approaches have been applied to these three categories of location predictions: the Markov chain model [103,104,105] and the trajectory pattern-based method which relies on frequent pattern mining (see Section 5.2.1) and the association rules among the patterns and corresponding influencing factors. For example, Ying et al. [106] first extracted trajectory patterns to identify the mobility behavior motivated by geographic, temporal, and semantic factors; they then matched the current movements of the objects of concern to the extracted patterns. Monreale et al. [107] introduced a decision tree called the T-pattern tree after extracting trajectory patterns as predictive rules. The tree was built and evaluated with a formal training and test process and eventually, shows a certain level of accuracy for next-location prediction.

In contrast, route prediction speculates a sequence of paths starting from a certain location of a certain moving object. This is generally conducted under strict built environment constraints (e.g., a road network) [108,109,110]. Currently, there are three approaches to route prediction: trip observation-based, Markov model-based, and turning behavior-based. Trip observation-based prediction is based on the fact that a large portion of a typical driver’s trips are repeated. Thus, this type of prediction utilizes observed locations of the object’s past trips to develop algorithms for end-to-end route prediction [110]. These algorithms essentially match the first part of an object’s current trip with its set of previously observed trips to determine the most likely following part. Markov model-based prediction is applicable to both location prediction and short-term route prediction. For route prediction, a simple Markov model is first trained from the object’s long-term trip history and then, applied to making a probabilistic prediction for the next road segment considering the path that the object just followed [111]. Turning behavior-based prediction is focused on the object’s turning choices at intersections. When strung together, these choices form a route in the road network. In other words, if the object’s aggregate turning behavior (including the choice to go straight ahead) can be predicted, its future route can be identified [112,113]. The Markov model is a typical method for turning behavior prediction, but other ways include pure statistical methods. For example, Krumm [113] proposed an algorithm and variations to infer the proportion of drivers that take each turning option at intersections based on the assumption that drivers are more likely to choose a turning option that offers more destination options.

### 5.3. Relationships between Trajectory Data Mining Methods

As discussed previously, first-tier methods are the foundation of second-tier methods. For most tasks, we need the former to categorize trajectories according to their homogeneity or heterogeneity, while the latter is used for deeper or more synthetic analysis on the already clustered or classified trajectories. Table 1 describes such relationship for specific tasks. Each task is concerned with a specific second-tier mining method, which naturally requires a corresponding first-tier method. For example, frequent pattern mining (second-tier) applies clustering (first-tier) to find places of significance [79,80]. There exists some overlapping between second-tier methods. For example, a prediction method (second-tier) can use pattern mining (also second-tier) to obtain a concise representation of the object’s moving behavior, which is essential for future location prediction [106,107].

## 6. Application Issues with Trajectory Data Mining

In the previous sections, we discussed three categories of emerging trajectory-related data and two classes of data mining methods for extracting information from trajectories. In this section, we look at the application issues that can be addressed with mining trajectory data. Current application issues can be sorted into three categories: social dynamics, traffic dynamics, and operational dynamics [6]. These issues can be matched with the trajectory data mining methods that were presented in Section 5. Note that there is no firm one-to-one mapping relationship between an application issue and mining method. A single application issue may require several mining methods, or different issues can be tackled with the same method. The matching relationships between issues and methods depend on the specific tasks involved with an issue. These relationships and their corresponding references are presented in Table 2, which may help guide other researchers to select the most suitable methods for specific application issues.

### 6.1. Social Dynamics Issues

To some extent, social dynamics can also be considered as community dynamics. A community is a group of entities that share some common interests. In the context of trajectory data mining, such interests are represented as common mobility behavior based on the observed trajectories [6]. In most cases, the application issues for social dynamics are not constrained by the built environment (e.g., road network). Rather, they are concerned with collective movement trends at the community, city, or even region level. This is in contrast to detailed network-based paths, which are motivated by various internal demands (e.g., work, shop, school) and affected by various external factors (e.g., weather, traffic, policy) [6,15]. Previous research on social dynamics issues has utilized trajectory data to tackle questions such as where people go during the day [15,137], the locations of hotspots (i.e., where traveling origins and destinations accumulate) around the city [138,139], the functions of these spots in an urban context [120,121], and the strength of connections between different parts of the city [140]. These studies have used diverse categories of trajectory data to reveal a well-rounded understanding of the urban reality. Zheng et al. [21] defined such data as digital footprints and the framework for mining trajectory data as urban computing, which they have detailed in several papers [4,21,22].

#### 6.1.1. Discovery of Social Relationships

Theoretically, trajectory data can be used to extract existing interactions between moving objects and discover more about the properties of these interactions. Such interactions and their properties are defined here as social relationships; they include those between individuals, communities, or even animals (e.g., predator-prey interactions [141]).

To what extent can interpersonal relationships be inferred from spatiotemporal trajectories? The wealth of geographic information in social media has provided an opportunity for researchers to explore this question in detail. For example, Crandall et al. [142] proposed two sub-questions: (1) Provided that, on multiple occasions, two individuals are in roughly the same geographic location at nearly the same time, how likely are they to know each other? (2) How does this likelihood depend on the proximity of the co-occurrences in time and space? They then established a framework for quantifying answers from a social media website and found that a high likelihood of interpersonal ties can be triggered, even from a small chance of co-occurrences. They also built a probabilistic model to show how such large probabilities of social ties arise from co-occurrences. Meanwhile, some researchers have attempted to answer this question by examining the relationship between individuals’ social ties and their visits to the same places [117,143]. For example, Wang et al. [143] tracked the trajectories and communication records of millions of cellphone users and discovered that the similarity between two individuals’ movements strongly correlates with their proximity in a social network.

In terms of community-level social relationships, many studies have followed classical clustering and pattern mining methodologies, as discussed in Section 5 [6,117,144]. For example, Gaito et al. [117] proposed the concept of a geocommunity, which combines the geolocations of individuals and social communities with common mobility interests. They extracted geolocations by clustering the stay-locations of individuals and then, utilized density-based clustering to discover their communities. They then adopted sequential pattern analysis methods to detect the social relationships between communities.

#### 6.1.2. Detection of Social Events

From the perspective of trajectory mining, a social event refers to a gathering of long-term but temporary stay-locations. Thus, detecting social events involves recognizing the existence of such gatherings. When combined with the properties of the gatherings (e.g., semantic information), the type of social event can also hopefully be identified [145]. Typically, there are three methods for recognizing social events from trajectories: statistics-based, classification-based, and clustering-based. In many cases, multiple methods are applied.

As a typical example of statistics-based detection, Giannotti et al. [130] discovered social events by identifying a high concentration of stationary objects that were previously moving within a specific spatiotemporal constraint. This method can be followed by a classification procedure to identify the event type. For example, Calabrese et al. [119] classified the feature vectors of attendees’ origins detected from cellphone data to estimate the type of event.

As a more realistic scenario, Zheng et al. [118] proposed the snapshot, which indicates a social event that satisfies the following conditions: (1) the groups of individuals are dense, (2) the shape and location of the groups generally do not change, and (3) the group members can enter and leave at any time as long as there are a certain number of members in this group for a certain period of time. They then proposed a density-based clustering method to detect snapshot clusters, from which gathering (i.e., social event) patterns can be extracted.

#### 6.1.3. Characterization of Connections between Places

The characterization of places and profiling of connections between places are closely related application issues for trajectory data mining. Both utilize origin–destination (OD) information as the key to uncovering urban realities at a relatively macro scale, and they share similar mining methods (e.g., hierarchy-based clustering, density-based clustering, classification).

Detecting hotspots within a city is a first-tier task for characterizing a place. A hotspot refers to a region where urban activities regularly accumulate [138,139] and is usually mined through clustering methods. For example, Chang et al. [138] considered areas with a high intensity of taxi requests to be a typical kind of urban hotspot and clustered passenger pick-up points (which can be distinguished in taxi GPS datasets based on certain field values) to discover such hotspots. They even built a hotness index based on the properties of these clusters. Liu et al. [146] also used a clustering-based approach to represent urban hotspots by certain crowdedness dynamics considering the real clustering properties of objects. They proposed a non-density-based approach called mobility-based clustering, where each sample object is utilized as a sensor to perceive the crowdedness around it by using its instant mobility properties (e.g., a taxi’s instant speed).

Another type of application is identifying the land use types and regional functions within a city. Such work is generally conducted in two steps: clustering to extract regions and classification to assign function-related properties to the extracted regions [120,121,147]. For example, Pan et al. [121] tried using taxi GPS trajectories to classify urban land use. They applied a modified density-based clustering method called iterative DBSCAN to extract regions. They then classified regions into different social functions based on the taxis’ pick-up and drop-off dynamics.

Similarly, characterizing connections between places also relies on the OD mechanism within the trajectories. For example, Liu et al. [122] utilized taxis’ pick-up and drop-off records (PDRs) and the check-in and check-out records generated by smartcards to study the connected regions and corresponding connection strength. They applied clustering techniques to analyze the trip relationship between different zones with an OD matrix. As a complement to such research, abnormal connections can be detected with outlier identification methods as discussed in Section 5.2.2, which discriminate abnormal connections from normal ones [124]. Another issue is identifying the properties of the detected anomalies, which, in turn, comes back to the above characterization of connections [148].

### 6.2. Traffic Dynamics Issues

Traffic dynamics specifically refers to how people carry out their mobility intentions depending on the road network or other built environments and governed by their underlying travel demands [6]. In this paper, we broaden the understanding of traffic dynamics issues by referring to tasks that are directly related to the movement or moving object, as well as predictions directly based on existing movements. Unlike research on social dynamics, which principally utilizes OD-related information, research on traffic dynamics usually makes full use of trajectories.

#### 6.2.1. Profiling of Moving Objects

Starting from the trajectory, the most direct type of research would be to profile the moving object that generates the trajectory. Such research includes but is not limited to deducing the activity types of humans [125], profiling the mobility routine of humans [127,128], inferring transportation modes [61,62], understanding the moving behavior of animals [126,149], and describing the movement patterns of animals [88,150]. These issues seem scattered but are essentially concerned with the inherent properties of the trajectories and have the common ambition of understanding the behavior of the moving object.

Research in this category has mostly been focused on inferring the activity types of travelers and identifying their traffic mode. These two issues can be addressed through classification methods based on some preset features, as done by Zheng et al. [62]. Clustering methods are used in frequent pattern mining to deal with profiling issues for human mobility routines and animal movement patterns. Such approaches essentially extract the sequences of places that moving objects have frequently visited, as discussed in Section 5.2.1.

#### 6.2.2. Trajectory-Based Prediction

In Section 5.2.3, we discussed prediction in detail as a major trajectory mining method. This involves two major issues: predicting the next position (or destination) of the moving object and inferring the route that the moving object will follow. Here, we introduce another major issue: forecasting the occurrence of traffic-related incidents such as traffic congestion. Specific solutions for the first two issues were presented in Section 5.2.3. Thus, here we only discuss the methods for predicting traffic congestion.

Areas with traffic congestion essentially have high traffic density. Thus, the problem of predicting congestion can be transformed into the problem of inferring traffic density. Giannotti et al. [130] established a tree structure formed by T-patterns to predict the locations of areas where large amounts of trajectories accumulate. Each T-pattern represents a sequence of visited positions and the corresponding transition time, and each tree node carries a support value indicating the number of T-patterns that connect the tree root to the current node. Another approach is extending the classical Markov-based route prediction method. The predicted routes will eventually constitute a certain level of traffic density. By comparing the predicted traffic density with the capacity of the corresponding road segments, we can theoretically find areas at risk of congestion. Castro et al. [131] used this approach to build a prediction model based on the probabilities of switching between road segments and determined the capacity by referencing the historical traffic density.

### 6.3. Operational Dynamics Issues

In this paper, operational dynamics refers to the information that can be extracted from trajectory data, which can potentially be applied to social, economic, commercial, or other operations. Compared with the above two categories, issues with operational dynamics require deeper mining to reveal the properties hidden within the trajectories.

#### 6.3.1. Interest Recommendation

Interest recommendation is based on the hypothesis that people who share similar mobility profiles are likely to share similar interests and preferences [132]. There also exists a two-way positive interaction mechanism between potential friendship and shared movement patterns. In other words, if two people consistently display similar mobility profiles, they can be recommended to become friends. In turn, if a person’s friends frequently visit certain places or follow certain routes, these places and routes can be recommended to the person. This has become one of the most fundamental hypotheses in current social network operations.

A common method for interest recommendation is to mine the mobility history for frequent patterns. For example, Li et al. [132] established a framework for a friend–place recommender system with three internal modules: mobility history representation, user similarity evaluation, and friend-place recommendation. In the first module, hierarchy-based clustering is performed on the stay-locations of each user to obtain their mobility history, which is then visualized in a hierarchical graph. In the second module, similar sequences of shared graph nodes are retrieved from all users’ graphs, which are then used to generate similarity scores for each user pair. In the third module, users are ranked according to their scores in relation to a given user. Those ranking relatively high can then be recommended as potential friends to the given user. Places can be recommended to the user by integrating supplementary data (e.g., POIs, semantic tags) with the mobility histories of these potential friends. Li et al.’s framework [132] has been widely accepted by researchers and followed in several studies [133,134]. For example, Zheng et al. [134] established a fully executable friend–place recommender system based on this framework. Additionally, there exists interest recommendation research using implicit data, which explores beyond friend–place recommendations. For instance, Amato el al. [151,152] described a recommender system on the basis of the interactions among users and generated multimedia content, which can support different social applications using proper customizations (e.g., recommendation of news, photos, art pieces, etc.).

#### 6.3.2. Trip Recommendation

Another issue in this category is trip recommendation. This differs from the route prediction discussed in Section 5.2.3 and Section 6.2.2; although trip recommendation also produces a sequence of places to be visited like route prediction, it does not specify the visiting order within the sequence. Similar to interest recommendation, trip recommendation also mines mobility history for frequent patterns with some customization to deal with users’ specific trip preferences. For example, Brilhante et al. [135] formulated TripBuilder, which abstracts each user’s mobility history as a chronologically ordered (annotated with the start and end times) sequence of POIs and then, profiles each user’s trip preferences based on the functional classification of the POIs. This framework utilizes the wisdom of the crowds to find personalized itineraries for a user, given their trip preferences and visiting time budget. Zheng et al. [136] built a complete system that can mine GPS traces to perform two kinds of trip recommendations: a generic one indicating the most interesting places and routes of a given region, and a personalized one that provides the user with places matching their personal preferences.

## 7. Trajectory Data-Based Services

The information and knowledge obtained from trajectory data mining are applicable to a wide range of services. Services are rooted in real life while being based on the solutions to the application issues discussed in Section 6. Note that there is no specific one-to-one mapping relationship between application issues and trajectory data-based services. In fact, their links are rather flexible depending on specific scenarios. Table 3 lists some examples of trajectory data-based services and their relation to the corresponding application issues.

### 7.1. Transportation and Urban Planning

Transportation services generally require the characterization of regions (e.g., OD distribution, hotspot distribution) and commuters (e.g., preferred means of transportation, spatiotemporal law of commuting). Occasionally, realworld transportation services also require prediction and trip recommendations. Transportation services include but are not limited to improving the driving experience [153,154,155], augmenting public transit services [156,157,158,183], and transportation planning and management [14,160,161].

In urban planning, trajectory data mining has two main functions: characterizing locations and characterizing the connections between locations. These two categories can help urban planners understand urban boundary evolution [162], plan urban infrastructure [163,164], assess the transportation system [165,166], etc.

### 7.2. Environment and Energy

Evaluating the pollution at different locations is a prerequisite for pollution mitigation. To this end, trajectory data mining can be used to characterize places and integrate their properties with supplementary data, e.g., air pollution data [21,167] and noise pollution data [168,169], in order to describe the pollution situations in different regions of the city.

With regard to energy, trajectory data mining is concerned with discovering energy-consuming patterns from a regional or individual perspective. This is related to characterizing places and profiling commuters. Researchers have utilized trajectory data to mine the movement patterns of energy-wasting vehicles [170], establish eco-driving feedback platforms [184], select locations for eco-car charging infrastructure [171], etc.

### 7.3. Social and Commercial Services and Public Administration

Social services are mainly related to profiling individual movement patterns, discovering social relationships, and recommending interests. Addressing these application issues can help facilitate social services, recommend potential friends [133,134,142], suggest places and routes [172,173,174], understand community life [175,176], etc.

Commercial services need information regarding the visiting potential of commercial places based on the mobility routines of consumers. Characterizing places and profiling individuals can help improve commercial services, such as optimizing commercial siting [177], guiding advertising allocation [178], and improving department layouts [179].

Public administration is often related to identifying places or individuals that are likely to trigger public incidents. Characterizing places and moving objects are significant in this domain. In addition, public administration requires a certain foresight regarding mobility dynamics; thus, trajectory-based prediction is also important. Researchers have improved public administration by detecting abnormal behavior [180], monitoring public events [181], monitoring and predicting hurricane movement [182,185], etc.

## 8. Practical Implications

In previous sections, we reviewed the concepts, methodologies, application issues, and resulting services of trajectory data mining from a rigorous academic perspective. In this section, we present an open discussion on the practical implications. For potential participants in the domain of trajectory data mining, a series of commonly used practical tools are recommended. For the most concerned privacy protection problem involved in this domain, a brief survey on current situations is conducted and potential solutions are proposed. There is also a future outlook based on our surveyed literature, which may indicate some directions for authors about trajectory data mining.

### 8.1. Practical Tools in Trajectory Data Mining

The practice of trajectory data mining methodologies requires the use of certain software tools. As the number of available tools continues to grow, it is increasing difficult to define a most suitable tool, or even to determine the most widely accepted tools nowadays. The typical life cycle of new tools generally begins with theoretical papers as methodological prototypes, followed by demand-responsive software distribution of successful algorithms [186]. These algorithms are either included as a family in new commercial or open-source packages, or are being integrated into existing commercial or open-source packages afterwards.

In fact, from the very beginning, programming languages provide researchers with the initial tools to conduct trajectory data mining. Python, for example, is a universal computer language that is widely applied in this domain [187]. If the users have become familiar with basic programming concepts such as variables, data types, functions, conditions, loops, etc., and are good at learning from online technical communities, such as GitHub, it is not very difficult to build up a basic data mining program. We always encourage researchers to be proficient in one of the computer languages (e.g., Python, C, Fortran), since this will enable them to easily convert algorithms or even algorithm thoughts into practice, without being restricted by the software platform.

Apart from pure programming languages, open-source software can be another good choice for researchers and learners in trajectory data mining. R is both a computer language and software that is powerful in statistical analysis [188]. Although its core computing modules are written in C, C++, and Fortran, it also provides a scripting language, i.e., R language, for customized programming. A series of analysis techniques, including statistical testing, predictive modeling, and data visualization, is supported by R. WEKA is another famous and powerful open-source software for data mining [189], which supports data preprocessing, data collection, classification, regression analysis, visualization, feature selection and many other machine learning functions. Advanced users can call its components through Java programming and command lines, while it also provides a graphical inference for basic users. KNIME is a platform that can be extended to use the mining algorithms in WEKA. Besides, it integrates many other data science tools covering data management, modeling, deploying, reporting, etc. [190]. KNIME uses a data flow-like approach to establish the mining process, which is composed of a series of functional nodes. Each node has an input port for receiving data and models, and an output port for exporting results, thus, users can easily connect to the nodes for process management. RapidMiner is also an extendable platform that can be applied in this domain. It owns a specific advantage in machine learning, by providing support for any third-party machine learning libraries [191].

Although the open-source software mentioned above can assist us in trajectory data mining, the power of commercial software can hardly be ignored. In order to obtain greater profits in a limited product life cycle, these tools are made more attractive in terms of user-friendliness and strong service support. IBM SPSS Modeler is a representative commercial tool for mining tasks. It is equipped with an intuitive user interface and allows users to create various algorithms without programming [192]. Oracle Data Mining (ODM) is a component of the Oracle platform, which is a world-famous database management tool. It enables users to build and apply models directly inside their Oracle Database [193]. SAS Data Mining is another commercial option with user-friendly GUI and specific strength in predictive modeling and prescriptive modeling [194].

Advances in computing power have enabled us to move beyond manual and time-consuming mining practice to quick and automated data analysis, meanwhile bringing about many powerful tools for trajectory data mining. Each tool has its own strengths. For scholars who need to dig deeply into the philosophy and methodology of data mining, we recommend programming languages and open-source tools with more customization possibilities. For business users who pursue practical efficiency and stable output, highly integrated commercial software may be a better choice.

### 8.2. Privacy Protection in Trajectory Data Mining

With the ubiquity of smart devices and the improvement of powerful data mining techniques, there are increasing concerns that trajectory data mining may pose a threat to our privacy and information security. However, we need to notify that the majority of applications in this domain are not deeply concerned with private information.

Consider a most extreme case that is happening at this moment: scientists are utilizing multi-source trajectory data to trace the transmission chain of COVID-19 (coronavirus disease). The major objective is to act quickly, when a person is diagnosed with COVID-19, to find all the people this person was in close proximity with [195]. One popular approach is contact tracing based on “check points” as suggested by Yasaka et al. [196], which uses an anonymized graph of interpersonal interactions to report risk levels to users. This process does not technically need any location information or personal data. Another approach is using Bluetooth-based smartphone apps, e.g., the TraceTogether app from the Singaporean government. Such apps cryptographically create a new temporary ID periodically and utilize Bluetooth’s near-field communication function to record IDs of close contact. If any user is diagnosed with COVID-19, the doctor will instruct them to share locally stored data with the central server. The server will obtain all the temporary IDs the “infected phone” has been in contact with, and then, inform them with a push token technique.

As indicated above, even under the conditions of a pandemic, we are still able to avoid privacy offences when taking advantage of trajectory data, typically with two ideas: the first is to represent personal information with virtual IDs during the process of tracing and publicity; the second is to replace absolute geographic coordinates with relative position information when unnecessary, e.g., when no mass infection is detected. In fact, apart from the emergent situations concerning individuals, the major focus of trajectory data mining is on the discovery of general or significant patterns, not on the specific information regarding individuals. For this reason, we believe that the real concerns are with unconstrained access to individual records, especially privacy-sensitive information such as religious, financial, or healthcare records that usually come along with implicit trajectory data. For applications that do involve such information, simple desensitization approaches, such as removing sensitive IDs from data, or to a more advanced degree, such as randomization methods [197] and encryption methods [198], are sufficient enough to protect the privacy of most individuals.

Nevertheless, privacy protection discussed here is from a pure technical perspective. In the real world, concerns cannot be completely eliminated whenever and wherever sensitive information is collected and stored in a digital form. Like any other technology, trajectory data mining is possible to be misused. Thus, not only the researchers in the general data mining domain, but also those in the fields of database encryption, counterterrorism, and social sciences, are expected to work with lawyers, politicians, entrepreneurs, and consumers to take responsibility in establishing solutions to protect personal privacy and data security.

### 8.3. Future Prospects for Trajectory Data Mining

The diversity of trajectory data, mining methods, and mining applications has brought about a series of challenging research issues. From the surveyed literature in this paper, we can glimpse some development trends in trajectory data mining and provide suggestions for authors in this filed.

The first significant trend is to combine trajectory data with other data sources to fulfil a mining task. The rationality of integrating multi-source heterogeneous data first lies in avoiding information bias, or in other words, enriching trajectory information with other sources. An example can be found in Wang et al. [199], which leveraged POIs and road network data to fill in the missing information in sparse trajectories, in order to better estimate the travel time of a path in a city’s road network. On the other hand, such combination may unlock the potential power of knowledge that can hardly be discovered from a single data source. For instance, Zheng et al. [200] inferred the fine-grained noise situation of different times of day for each region of New York City by using the 311 complaint data together with social media check-in data, road network data, and POIs.

The second prospect in this field is the development of scalable and interactive mining methods. Unlike traditional data analysis, data mining must be able to process huge amounts of data effectively, and if possible, interactively. As the amount of data increases rapidly, it is essential to develop more scalable algorithms for mining tasks. The incremental trajectory clustering algorithms in Li et al. [51], for example, are an early-stage attempt to deal with such dynamic data growth. To this end, Ding et al. [201] established a united platform named Ultraman to achieve scalability and efficiency when dealing with big trajectory data, by extending Apache Spark with an integrated key-value store and enhancing the MapReduce paradigm to allow flexible optimizations based on random data access. Practical direction to improve the overall efficiency and user interaction is constraint-based mining. As integrated in many practical tools such as KNIME [190], it endows users with added control by allowing specifications and constraints to guide the mining workflows in their search for interesting knowledge.

Beyond trajectory data mining itself, privacy handling is another task to be tackled in the future. There exists an underlying balance between a sufficient degree of useful knowledge and the ethics of tracking activities. Although there have been many technical approaches to protect personal privacy as mentioned previously (e.g., randomization [197] and encryption [198]), no promising methods to achieve or even measure this balance have yet been developed. In addition, the best results with real-life relevance of trajectory data mining can be achieved by interdisciplinary efforts. This community is expecting more research with extensive practical significance and interdisciplinary influence.

### 8.4. Trajectory Data Mining in Industry 4.0

Recent decades have witnessed that people and things are becoming increasingly interconnected. Smartphones, vehicles, devices, built environments, and natural environments have been filled with digital sensors, all of which are generating unprecedented big data, including trajectory data as we have discussed. Urban realities mined from such data make it possible that demand-responsive urban services be highly realized. For instance, the digitization journey in the transportation and logistics sector is already well underway and is expected to accelerate in the immediate future. Companies are using IoT solutions, such as big data analytics, for demand forecasting and, in turn, optimizing inventory planning, warehousing fulfillment, and distribution [202]. The consequence of developing such IOT solutions and Big Data science is the conception of Industry 4.0 [203].

Industry 4.0 refers to the fourth industrial revolution. Its initiative places significant emphasis on the utilization of data to form intelligent systems and processes, so that in this context, manufacturing and service-providing will largely have the ability to self-plan and self-adapt [203]. To accomplish the vision of Industry 4.0, or even to survive in the “Digital Darwinism”, digital transformation is a common task faced by individuals, enterprises, and countries [204]. Yet, even in highly integrated Europe, a digital divide does exist among the member states [205]. Northern Europe takes the lead in terms of the utilization of innovative industries, such as the application of big data, while there also exists a worrisome trend of the European countries lagging behind other global leaders of Industry 4.0, such as the USA and China [205]. Our literature survey on trajectory data mining, to some extent, has also confirmed this trend, as the majority of research cases in this specific domain are contributed by countries with Industry 4.0 advantages.

In the new digital divide, Industry 4.0 will play an important role for individuals, in that routine jobs are likely to be replaced by those requiring analytical skills, flexibility in decision making, and training in certain topics, such as trajectory data mining, text mining, and machine learning, as proved by many recent surveys on Industry 4.0 employment demands, for example, in Bach et al. [206] and Fareri et al. [207]. Regardless of the social skill requirements of Industry 4.0 job positions, trajectory data mining is of educational advancement from a technical perspective. Not only can it address the specific application issues mentioned previously, it also provides a methodology for data evidence-based decision making, which will largely benefit future participants in Industry 4.0. Therefore, the practical implications here indicate the need for interventions of education, such as curriculum with a focus on big data acquisition, management, mining, and analysis.

## 9. Conclusions

Advances in smart infrastructure and location-acquisition terminals have contributed to the increasing availability of massive trajectory data with rich spatiotemporal information on the mobility of a wide range of moving objects, including humans, vehicles, and animals. By developing data mining and analysis methods, researchers have revealed abundant urban realities from trajectory datasets, such as the movement patterns of humans, the inter-place relationships within cities, and the dynamics of social events to solve complex urban problems in transportation, environment, public security, etc.

However, despite the wealth of information in this field, existing studies have been relatively isolated and lacking an integrated and systematic survey to the issues that have been addressed, solutions that have been tested, and services that have been developed. This paper was an attempt at conducting such a survey. We started with classifying diverse trajectory data and reviewed the prevailing trajectory mining methods in two classes. We classified the application issues of trajectory data mining into three major groups based on how they are related: social dynamics, traffic dynamics, and operational dynamics. We then built up matching relationships between data mining methods and application issues, and briefly presented the prospects of services that have been established based on the methods and techniques in this field. A series of open discussions was also conducted regarding the practical tools, ethics, and future directions of trajectory data mining.

The major contribution of this paper is in providing a systematic and integrated view on the emerging issues, methodologies, and services in trajectory data mining while identifying the inherent relevance and associations that have previously been unrepresented in this domain. The classification of application issues can help readers to identify new issues where trajectory data mining could be applied. The relevance between mining methods and the matching relationship between mining methods and application issues can contribute to identifying method gaps and inspire researchers to develop new methods. The consistent association linking methods, application issues, and services can provide a reference for data analysts and experts to select the most suitable solutions for specific problems. This paper can also provide new researchers with a quick understanding of trajectory data mining.

The main limitation of our work lies in the manual selection of surveyed literature, which may lead to certain information bias. Besides, our work is based on the review of the articles, reports, and book sections that can be accessed through Web of Science at present. Due to the time difference between literature creation and publication, as well as the imperfect search function, specific research, e.g., the latest ones or those Web of Science has no authorization to disclose, might be invisible in our review.

The smallest unit of analysis in our work is the application-oriented method. In fact, within each method, there correspondingly exist a series of algorithms. Therefore, our work can be extended by conducting a comparative analysis on the algorithms that implement a specific mining method on specific application issues, so that the review will be able to provide a reference for the choice between algorithms. Additionally, research work concerning the future prospects we have discussed in the section of practical implications may also be promising under the current context.

## Figures and Tables

**Table 1:** Relationships between trajectory data mining methods.

	Categories	First-Tier Mining Methods	
Categories	Methods	Clustering	Classification
Second-tier Mining Methods	Pattern Mining	Grouping spatially close trajectories [86,87]; Grouping temporally related trajectories for periodic pattern mining [69,71,73]; Extracting places of significance for frequent pattern mining [79,80]; Detecting similar mobility interests for collective pattern mining [83,86,87,88,89]; Aggregating close locations for sequence analysis [114];	No classification-related tasks have been identified for pattern mining.
	Outlier Identification	Grouping trajectories or sub-trajectories with homogeneity [91,94];	Sorting out trajectories based on pre-identified features [95,96];
	Prediction	Grouping multiple users with similar mobility intentions [102,115]; Grouping similar trips of one specific object [116]; Mining trajectory patterns for location prediction [100,101,106,107];	Matching one object’s current movement with its movement patterns for location prediction [107,116]; Matching one object’s ongoing trajectory with its previous trajectories for route prediction [110];

Note: Not all cases are listed. Information in this table is summarized based on our literature survey.

**Table 2:** Relationships between trajectory-related application issues and trajectory mining methods.

Application Categories	Application Issues	Description of Issues	Major Tasks Involved	Mining Methods Involved
Social Dynamics	Discovery of Social Relationships	Discovery of social ties between individuals and communities	Grouping individuals’ stay locations	Clustering [117]
			Extracting chronologically ordered sequences of stay locations	Frequent pattern mining [117]
		Discovery of interaction between animals	Detecting groups of moving animals, describing groups’ features	Collective pattern mining [88]
	Detection of Social Events	Detection of event occurrence	Grouping based on spatiotemporal properties	Clustering [118]
		Profiling of discovered events	Extracting features and categorizing events	Classification [119]
	Characterization of Connection between Places	Detection of hotspots	Grouping according to spatiotemporal properties	Clustering [120,121]
		Description of land uses and regional functions	Discovering regions with similar functions	Clustering [120,121]
			Extracting features and categorizing regions	Classification [120,121]
		Description of connection between places	Extracting origin/destination links	Clustering [122,123]
			Discriminating abnormal links	Outlier identification [124]
Traffic Dynamics	Profiling of Moving Objects	Inferring mobility activities and modes	Extracting features and categorizing activities	Classification [61,125,126]
		Profiling movement patterns	Extracting sequences of visited places	Frequent pattern mining [127,128]; Clustering [129]
	Trajectory-based Prediction	Predicting an object’s future location/route	Establishing probabilistic model for prediction	Statistical methods (e.g., Markov Chain) [104,105]
			Comparing current trajectory with extracted historical trajectories	Frequent pattern mining [106,107]
		Predicting traffic jams	Inferring traffic density and comparing it with road capacity	Frequent pattern mining [130]; Statistical methods [131]
Operational Dynamics	Interest Recommendation	Friend–place recommendation	Extracting shared movement patterns and ranking similarities	Frequent pattern mining [132,133,134]
	Trip Recommendation	Suggesting order of visiting locations	Predicting routes based on user preferences	Frequent pattern mining [135,136]

Note: Information in this table is summarized based on our literature survey. Application categories are recommended by Castro et al. [6].

**Table 3:** Relationships between trajectory data-based services and application issues.

Services	Service Contents	Application Issues Involved		
		Social Dynamics	Traffic Dynamics	Operational Dynamics
Transportation	Improving driving experience			Trip recommendation [153,154,155]
	Augmenting public transit services	Characterization of connections between places [96,156]	Trajectory-based prediction [157,158,159]	
	Enhancing transportation planning and management	Characterization of connections between places [14,160]	Trajectory-based prediction [161]	
Urban Planning	Understanding urban land use and urban evolution	Characterization of connections between places [120,162]		
	Facilitating urban infrastructure planning	Characterization of connections between places [163,164]		
	Evaluating transportation system	Characterization of connections between places [165,166]		
Environment	Assessing air pollution	Characterization of connections between places [167]		
	Assessing noise pollution	Characterization of connections between places [168,169]		
Energy	Inferring energy consumption	Characterization of connections between places [170]		
	Eco-car infrastructure planning	Characterization of connections between places [171]	Profiling of moving objects [171]	
Social Services	Supporting friend-searching	Discovery of social relationships [133,142]	Profiling of moving objects [133,142]	Interest recommendation [134,142]
	Suggesting routes and places		Profiling of moving objects [172,173,174]	Trip recommendation [172,173,174]
	Understanding communities	Discovery of social relationships [175]	Profiling of moving objects [175,176]	
Commercial Services	Optimizing commercial localization	Characterization of connections between places [177]	Profiling of moving objects [177]	Trip recommendation [177]
	Guiding advertising allocation	Characterization of connections between places [178]	Profiling of moving objects [178]	Trip recommendation [178]
	Optimizing department layout	Characterization of connections between places [179]	Profiling of moving objects [179]	
Public Administration	Detecting abnormal behavior		Profiling of moving objects [180]	
	Monitoring public gathering	Detection of social events [181]	Profiling of moving objects [181]	
	Predicting natural disasters		Trajectory-based prediction [182]	

Note: Information in this table is summarized based on our literature survey. Not all cases are listed.
